# Can grimace scales estimate the pain status in horses and mice? A statistical approach to identify a classifier

**DOI:** 10.1371/journal.pone.0200339

**Published:** 2018-08-01

**Authors:** Emanuela Dalla Costa, Riccardo Pascuzzo, Matthew C. Leach, Francesca Dai, Dirk Lebelt, Simone Vantini, Michela Minero

**Affiliations:** 1 Dipartimento di Medicina Veterinaria, Università degli Studi di Milano, Milan, Italy; 2 MOX Laboratory for Modelling and Scientific Computing, Department of Mathematics, Politecnico di Milano, Milan, Italy; 3 Newcastle University, School of Natural and Environmental Sciences, Newcastle upon Tyne, United Kingdom; 4 Equine Research & Consulting, Inca, Spain; University of Bari, ITALY

## Abstract

Pain recognition is fundamental for safeguarding animal welfare. Facial expressions have been investigated in several species and grimace scales have been developed as pain assessment tool in many species including horses (HGS) and mice (MGS). This study is intended to progress the validation of grimace scales, by proposing a statistical approach to identify a classifier that can estimate the pain status of the animal based on Facial Action Units (FAUs) included in HGS and MGS. To achieve this aim, through a validity study, the relation between FAUs included in HGS and MGS and the real pain condition was investigated. A specific statistical approach (Cumulative Link Mixed Model, Inter-rater reliability, Multiple Correspondence Analysis, Linear Discriminant Analysis and Support Vector Machines) was applied to two datasets. Our results confirm the reliability of both scales and show that individual FAU scores of HGS and MGS are related to the pain state of the animal. Finally, we identified the optimal weights of the FAU scores that can be used to best classify animals in pain with an accuracy greater than 70%. For the first time, this study describes a statistical approach to develop a classifier, based on HGS and MGS, for estimating the pain status of animals. The classifier proposed is the starting point to develop a computer-based image analysis for the automatic recognition of pain in horses and mice.

## Introduction

Pain assessment is a fundamental component of the effective safeguarding of animal welfare and practitioners working with animals require valid tools to assess pain to guide their pain management decisions [1,2]. The accuracy of pain assessment scales is directly dependent on their validity and reliability. These two concepts are inter-dependent as a scale cannot be valid without also being reliable: validity ensures the scale is measuring pain and reliability guarantees that it is measured consistently. Validity studies should confirm that the scale is able to differentiate animals “in pain” due to a painful condition from animals that are not experiencing pain or those experiencing other states, such as fear, anxiety etc. Expanding the concept of validity, a data-driven statistical procedure in which a pain status is estimated is defined as classification rule. A classification rule (i.e. classifier) is a tool to predict which of several distinct groups any new observation belongs to, based on a training set of data containing observations whose group membership is known. An example would be whether, based on a pain scale, it is possible to infer the pain status of a given animal. Statistical classification techniques also represent an essential tool to investigate whether different observers can reliably use a pain scale to assess the pain condition. This is particularly relevant in veterinary practice where pain scales rely on the recognition of a limited number of signs by different assessors, who could be subject to some degree of variability relating to differences in perception, clinical experience and knowledge of the species behaviour [3].

In humans, facial expressions are considered a reliable indicator in neonatal pain assessment [4–7] and recently a neonatal pain expression recognition method utilising a classifier has been proposed [8]. The main reason given for using a classifier was to assist the clinicians in assessing neonatal pain by utilising computer-based image analysis techniques. An automatic monitoring system, based on a classification paradigm, was also proposed for the pain recognition in patients with shoulder pain [9–11]: with this aim a shared database was created and consisted of 200 video sequences containing spontaneous facial expressions of pain and associated pain frame-by-frame scores [12]. A classifier for monitoring facial expressions could increase the reliability, sensitivity, and precision of the research into the relationship between facial signs and experiences of pain, and so lead to new insights and diagnostic methods [13–15]. Facial expressions have been investigated for pain assessment in several non-human mammal species, including mice [16–18] and horses [19–21]. The Horse Grimace Scale (HGS) [19] consists of the sum of the following six Facial Action Units (FAUs): stiffly backwards ears, orbital tightening, tension above the eye area, prominent strained chewing muscles, mouth strained and pronounced chin, and strained nostrils and flattening of the profile. The Mouse Grimace Scale (MGS) [16] is the sum of five facial features (FAUs): orbital tightening, nose bulge, cheek bulge, ear position, and whisker change. Both HGS and MGS have been partially validated for pain assessment under different conditions [15–19, 21, 22]. HGS was applied in post-surgical condition [19] as well as orthopaedic pain [20]; MGS was used in experimental models of pain [16] and post-surgery [18,23]. Further validation of both HGS and MGS is critical for investigating the accuracy of the method in classifying when an animal is in pain prior to applying in a clinical setting. Furthermore, there remains little or no indication about the relative role (or weight) of different FAUs in pain recognition for either HGS and MGS, meaning that some FAUs could be more related to pain condition than others. In terms of inter-observer reliability, HGS and MGS seem to be easily and effectively applied by new assessors after a short training with a good degree of accordance [17,19,20]. As far as we are aware, there have not been studies reporting the development of a classifier to improve pain recognition of a given animal using either of these grimace scales.

The use of partially validated pain scales to assess animal pain could represent a serious threat to their welfare, consequently this study is intended to further progress in the validation of these grimace scales. We propose a statistical approach to identify an accurate and interpretable classifier that is able to estimate the pain status of the animal based on the FAUs included in the HGS and MGS. To this aim, we investigate, through a validity study, the relation between FAUs included in HGS and MGS and the real pain condition.

## Methods

This study details the analysis of previously published data on the HGS [19] (S1 Table) and MGS [18] (S2 Table) using a new statistical approach and interdisciplinary interpretation of results. The data consists of scores attributed by different observers to images collected in research settings of same animals (horses and mice) when pain free (baseline) and directly following painful surgical procedures (i.e. castration and vasectomy). The Horse Grimace Scale data [19] was collected from a study that was carried under the European Communities Council Directive 1986 (No. 86/609/EEC) and was registered with the Brandenburg State Veterinary Authority (V3-2347-A-42-1-2012). The Mouse Grimace Scale data [18] was collected from a study that was carried out under project and personal licenses approved by the Secretary of State for the Home Office, under the United Kingdom’s 1986 Animals (Scientific Procedures) Act and the Animal Welfare and approval from the Animal Welfare Ethics Review Board at Newcastle University. Procedures adopted for collecting data were all performed in accordance with relevant welfare guidelines and regulations. Two datasets, one for HGS and one for MGS, were separately analysed using Cumulative Link Mixed Model (CLMM), Inter-rater reliability (IRR), Multiple Correspondence Analysis (MCA), Linear Discriminant Analysis (LDA), and Support Vector Machines (SVM).

### Horse Grimace Scale (HGS)

Thirty-nine horses were assessed before and after routine castration under general anaesthesia, six subjects acted as anaesthetic only controls (for further details see Dalla Costa et al. [19]). Briefly, a total of 126 images of horses (63 pre-surgery and 63 post-surgery) were scored using the HGS by five treatment- and condition blind observers (equine practitioners). This gave a total of 630 image scores. For each image, each observer assigned a value of 0, 1, or 2 for each of the six FAUs (For further details see Dalla Costa et al. [19,24,25]). A score of 0 indicated high confidence of the observer that the action unit was absent. A score of 1 indicated either high confidence of a moderate appearance of the action unit or equivocation over its presence or absence. A score of 2 indicated high confidence of a marked appearance of the action unit. Therefore, the possible maximum HGS score was 12 (score of two for each action unit). For this study we selected a subset of 294 image scores taken from horses before and eight hours post- procedure (45 horses, of which 39 underwent surgical castration, and the other 6 underwent non-invasive indolent procedures under general anaesthesia) that matched the following criteria: both paired-images of the same horse before and after the procedure must have complete scores for each FAU given by the same observer. Horse image scores were divided in two groups: Pain (scores of horse images taken eight hours post-castration; N = 130) and No Pain (scores of horse images taken before the procedure, N = 147, and scores of horse images taken eight hours after the non-invasive procedure, N = 17).

### Mouse Grimace Scale (MGS)

Eighteen mice were assessed before and after scrotal approach vasectomy and either received saline, meloxicam (20 mg/kg sc) or bupivacaine (5 mg/kg li). For further details see Leach et al. [18]. Briefly, twenty-one treatment and condition blind observers with different levels of experience of mice (layperson, student, veterinarian, technician, scientist) scored 36 images (18 pre-surgery and 18 post-surgery) using the MGS. For each image, each observer assigned a value of 0, 1, or 2 for each of the five FAUs (For further details see Leach et al. [17]). A score of 0 indicated high confidence of the observer that the action unit was absent. A score of 1 indicated either high confidence of a moderate appearance of the action unit or equivocation over its presence or absence. A score of 2 indicated high confidence of a marked appearance of the action unit. From these images whisker position proved to be difficult to score (due to insufficient image quality) and so the FAU “whisker position” was excluded prior to any analysis [18]. The exclusion of whisker position is discussed in more detail in the original study [18]. Therefore, the possible maximum MGS score was 8 (score of two for each of the 4 remaining action units). From a total of 756 image scores we selected 638 image scores (319 pre- and 319 post-procedure) matching the following criteria: both paired-images of the same mouse before and after the procedure must have complete scores for each FAU given by the same observer. Post-procedure image scores were divided in three groups according to the treatment received by the mouse photographed: Post-Bupivacaine (N = 106), Post-Meloxicam (N = 103) and Post-Saline (N = 110). The Post-Saline group acted as a control for analgesia as they received a saline subcutaneous injection (2 ml/kg) administered 30 minutes prior to surgery.

### Statistical analysis

Cumulative Link Mixed Model (CLMM) [26] was used to assess how the pain condition of the horses (i.e., “Pain” or “No Pain”) or the pain condition with treatment (i.e., “Pre-op”, “Post-Bupivacaine”, “Post-Meloxicam” and “Post-Saline”) received by the mice (fixed effect), the observers and the subjects (random effects) influence the score of each FAU (response). This method is equivalent to an ANOVA model with mixed (1 fixed and 2 random) effects where the response variable is ordinal (i.e., the scores “0”, “1”, or “2”). In detail, CLMM is based on linear links between the effects and the logit transformations of the cumulative probabilities of assigning a score (“0”, “1” or “2”) to each FAU. By contrast to classical ANOVA for continuous response, CLMM considers and exploits the natural order of the FAU scores by the cumulative probabilities, without imposing an artificial constraint on the FAU scores which would derive from treating them as discrete numbers (i.e., assuming that category “1” lays exactly in the middle between categories “0” and “2”). Instead, similarly to the ANOVA mixed model, CLMM allows also the random effects to be included in the analysis. We assessed the significance of each effect (fixed and random) by a likelihood ratio test, comparing the full model with the reduced one, adjusting the p-values for multiple comparisons through the Benjamini-Hochberg correction to control the false discovery rate (i.e., the expected ratio between false positive results and positive results). We performed this analysis using the “ordinal” R package [27].

Inter-rater reliability (IRR) was evaluated with a two-way random, absolute-agreement, average-measures intra-class correlation coefficient (ICC), providing also correspondent 95% confidence intervals [28], in order to assess agreement among the observers in scoring each Facial Action Unit. We use the following guidelines to interpret the ICC measures [29]:

- less than 0.40: poor;
- between 0.40 and 0.59: fair;
- between 0.60 and 0.74: good;
- between 0.75 and 1.00: excellent.

This analysis was performed using the “irr” R package [30].

Multiple Correspondence Analysis (MCA) [31] was used to evaluate the overall relation within the FAUs scores, and among the FAUs and the pain condition of the horses (i.e., “Pain” or “No Pain”) or the treatment of the mice (i.e., “Pre-op”, “Post-Bupivacaine”, “Post-Meloxicam” and “Post-Saline”). MCA is an extension of simple correspondence analysis, a statistical method used to examine the co-occurrences between two categorical variables in a contingency matrix. MCA generalizes this framework, allowing several categorical variables to be considered, representing data in a sub-dimensional space. Therefore, this method can be viewed as the counterpart of PCA for categorical variables. It is also worth noting that the percentage of variance explained by the first dimension of MCA is severely underestimated [32], thus a correction formula (i.e., Greenacre’s correction) is applied to compute the appropriate percentage of variance explained. This analysis was performed with the “FactoMineR” R package [33].

### Classification analysis

Linear Discriminant Analysis (LDA) and Support Vector Machines (SVM) were used to classify the pictures in two groups according to the pain condition (“Pain” vs “No Pain”), based on the scores given by the observers.

LDA finds the linear combination of FAU scores that maximizes the discrimination between the two groups (the ratio between the “between-group” total variance and the “within-group” total variance) and computes an optimal threshold for the linear combinations for assigning units either to the first or to the second group [34]. It provides a simple interpretation in terms of the optimal weights to assign to each FAU score in order to achieve maximum classification accuracy. Such accuracy perfomance of LDA is assessed by estimating the probability of correct predictions through a leave-one-subject out cross-validation (LOSOCV) procedure, in which all the pictures of the same subject are removed successively (one subject at a time) from the training sample and then used for validation. Moreover, we find the best subset of FAUs to discriminate the “Pain” and “No Pain” groups with LDA by selecting, among the possible subsets of FAUs, the one that maximises the classification accuracy of LDA by LOSOCV.

SVM is a routinely used classifier from the field of machine learning, widely applied in biological, engineering, and other sciences [35]. It maps the original data features (or “training set”) into a higher-dimensional space through a kernel function (a non-linear transformation). Then, it finds the best linear separation of the two groups in this new space, seeking the Maximum Margin Hyperplane (MMH), the hyperplane (e.g. in 2D it is a straight line, in 3D a plane) that has the largest margin between the two classes. The main advantage of SVM is that the non-linear transformation performed at the beginning potentially allows linear separation of the groups in the new space, when this is impossible in the original space. This non-linear transformation can be chosen from among the most common alternatives, such as Gaussian Radial Basis Function, Laplace Function, and Polynomial Functions. Each kernel has different parameters that have to be tuned to identify the MMH, and we perform this tuning through a grid search approach. For the sake of completeness, we assess also the performances of a SVM with a linear kernel, which does not map the data into a higher dimensional space prior to find the MMH. Thus, among all the combinations that we explored with the grid search, we choose the optimal kernel function and its optimal parameters such that the probability of correct predictions is maximised. We computed such probability through a LOSOCV procedure with equal prior probabilities of belonging to the “Pain” or “No Pain” group, as it is done for LDA.

SVM is more flexible with respect to LDA, because it can be applied to different case studies with complex datasets. On the other hand, LDA has a far more intuitive interpretation of the results in terms of the weights of the features, as stated above. Instead, the determination of the MMH in SVM does not provide an explicit weighting of the features: SVM results in a complex black box model, the complex data transformations and resulting boundary surfaces are difficult to interpret. For these reasons, we propose LDA as the classifying method and SVM as purely benchmark method to compare the accuracy performance achieved through LDA. These analyses are performed with the “MASS” and “kernlab” R packages, respectively for LDA and SVM [36,37].

## Results

### Horse Grimace Scale (HGS)

The adjusted p-values of the CLMM are presented in Table 1. A significant association between the Pain group (Castration or Control) fixed effect and the scores is found for all the FAUs (all p-values were far below P<0.05), resulting in statistically higher scores for all the FAUs of the Castration group compared to Control group. A significant association between the observer random effect and the scores is found for mouth strained and strained nostrils (P = 0.0496 and P = 0.0524, respectively). A significant association between the horse random effect and the scores is found for all the FAUs (all p-values below P<0.05). Overall, the HGS demonstrated an “excellent” [29] inter-observer reliability with an average ICC value of 0.8560 (95%-confidence interval: [0.7810; 0.9060]). ICC values on average scores for each FAU and correspondent 95%-confidence intervals are reported in Table 2. Stiffly backwards ears, orbital tightening, tension above eye area, and prominent strained chewing muscles showed an “excellent reliability” with average ICC values ranging from 0.83 to 0.97. “Good reliability” was demonstrated for mouth strained (ICC = 0.6983) and strained nostrils (ICC = 0.6126).

**Table 1:** Cumulative Link Mixed Model (CLMM) on HGS dataset. Results from CLMM investigating the effects of Pain group, Observer and Horse on Facial Action Units. Adjusted p-values with Benjamini-Hochberg correction in relation to the respective category are reported for each FAUs. Correspondent likelihood ratio test statistics of the CLMM on HGS data are reported in brackets.

Facial Action Unit	Pain group	Observer	Horse
Stiffly backwards ears	<0.0001 (87.2780)	0.9988 (0.0003)	<0.0001 (88.9998)
Orbital tightening	<0.0001 (65.4123)	0.9988 (<0.0001)	<0.0001 (27.9243)
Tension above the eye area	<0.0001 (64.7438)	0.5771 (0.4279)	<0.0001 (38.3209)
Prominent strained chewing muscles	<0.0001 (53.9066)	0.4279 (0.8498)	<0.0001 (34.9081)
Mouth strained	<0.0001 (38.6054)	0.0496 (4.4065)	0.0359 (5.0988)
Strained nostrils	0.0002 (12.6588)	0.0524 (4.1861)	0.0299 (5.5683)

**Table 2:** Results regarding the inter-observer reliability analysis on HGS. Average scores of ICC, with the 95%-confidence interval reported in brackets, are presented for each FAU of HGS. Compared to Dalla Costa et al.[19], this analysis was carried out on a different sample of pictures as reported in the methods.

Facial Action Unit	Intraclass Correlation Coefficient (ICC)
Stiffly backwards ears	0.9655 (0.9528–0.9756)
Orbital tightening	0.8486 (0.7879–0.8962)
Tension above the eye area	0.8342 (0.7597–0.8910)
Prominent strained chewing muscles	0.8625 (0.8070–0.9056)
Mouth strained	0.6984 (0.5777–0.7928)
Strained nostrils	0.6126 (0.4511–0.7379)

**Fig 1:**
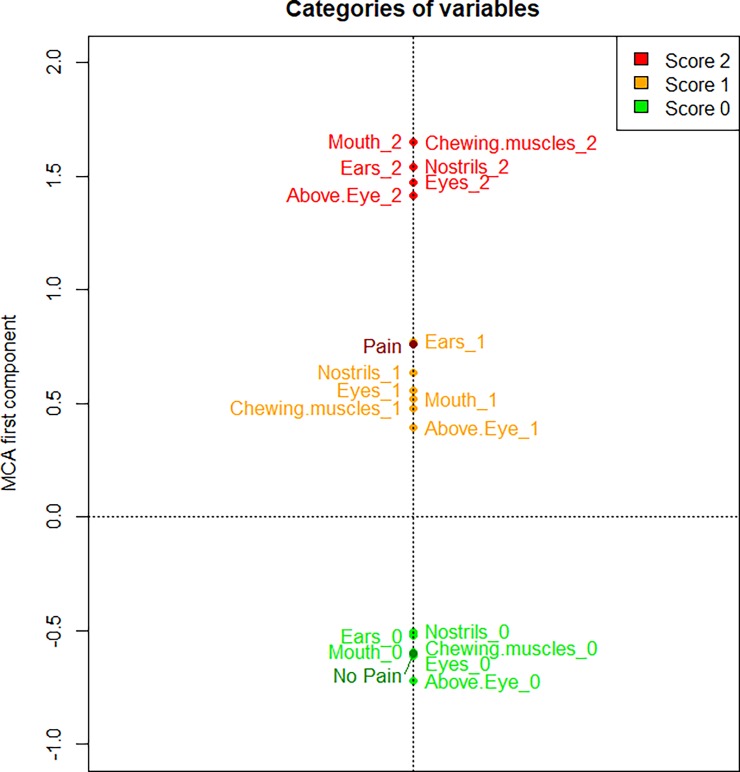
MCA results reported on the first component for HGS. The scores (values '0', '1' and '2') of the six FAUs of the HGS and the pain status (“Pain” vs “No Pain”) are presented, as average values, on the first component of the MCA.

**Fig 2:**
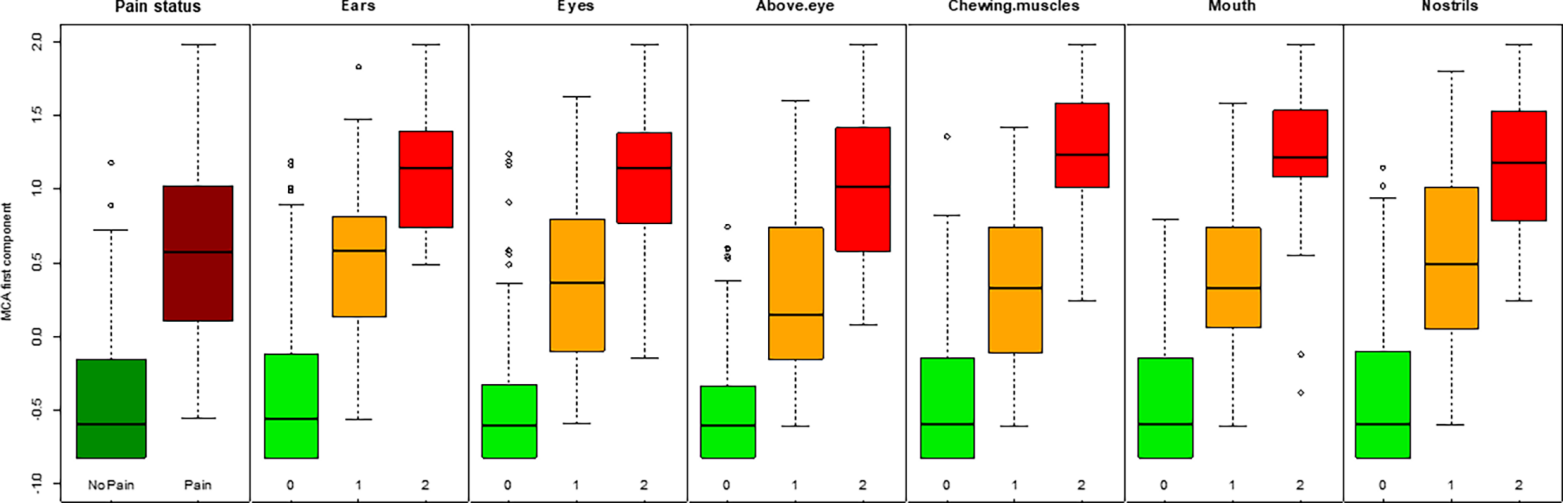
Boxplots of MCA first component scores stratified by pain group and FAUs score. The boxplots represent the dispersion of the MCA first component scores of the horse pictures, stratified by pain status ("No Pain", "Pain") and by score ("0", "1" and "2") of each FAU.

The MCA of the FAUs scores identified the first component (a one-dimensional space) to accurately describe the data, explaining a very high proportion of variation (90.73% computed according to Greenacre’s correction). The first MCA component is represented in Fig 1, where we plotted the average value of the component for the images of horses with pain and without pain (the labels “Pain” and “No Pain”, respectively) and the average value of the component for the images that have the same score for a FAU (e.g., the label “Ears_2” represents the average position on the MCA component of all the images having score 2 for stiffly backwards ears). Proximity in space between categories of different features indicates categories which are more likely to be observed simultaneously. This MCA component contrasts categories related to higher level of pain (positive values of the component in Fig 1) with categories related to absence of pain (negative values of the component). The intensity (score given) of all the six different FAUs are related, meaning that there are three well separated and equidistant groups of categories, corresponding to the scores (0, 1, or 2). The group “score 0” (all the labels of the FAUs with a score 0 in Fig 1) is highly related to the category “No Pain” (as they are closer in Fig 1), whereas the category “Pain” is related to the group “score 1” (all the labels of the FAUs with a score 1), and closer to group “score 2” (all the labels of the FAUs with a score 2 in Fig 1) than group “score 0”. This result is confirmed also looking at the variability of the scores of the first MCA component (Fig 2), where the horse pictures are represented and stratified by pain status and by score of each FAU.

**Fig 3:**
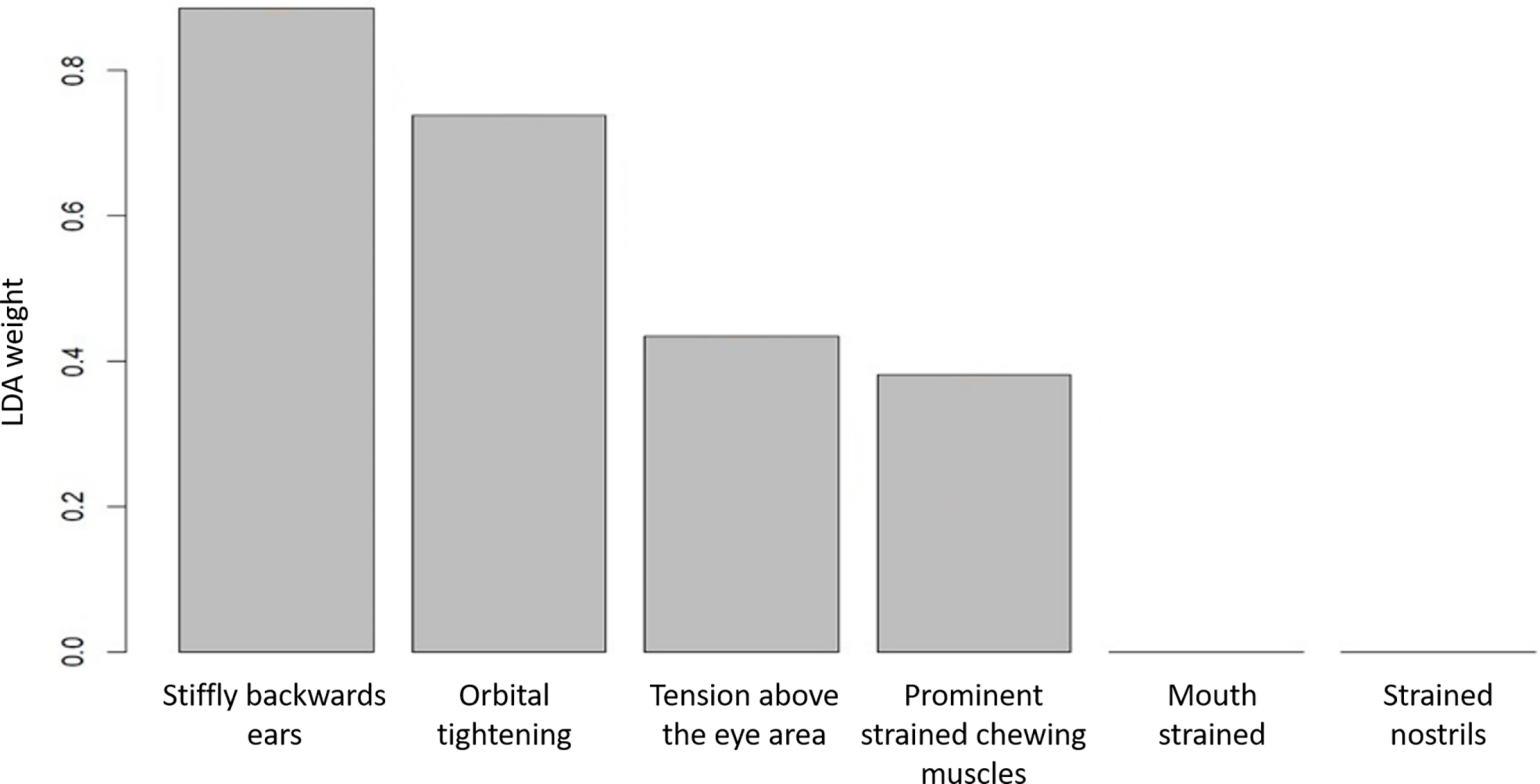
Weights of variables in the LDA component for HGS. Weights for each FAUs of HGS that best discriminate between the “Pain” and “No Pain” groups.

The LDA identified the weights of the FAU scores that can be used to best discriminate the “Pain” group from the “No Pain” (Fig 3), with a 77.4% of accuracy assessed by a LOSOCV procedure. Among the 294 scores analysed, LDA correctly classified 139 scores related to the “No Pain” group and 91 related to the “Pain” group, while 25 were wrongly assigned to the “No Pain” group and 39 to the “Pain” group. Scores for stiffly backwards ears, orbital tightening, tension above the eye area, and prominent strained chewing muscles contributed to the classification of the horses in the correct category (“Pain” vs “No Pain”). Scores for mouth strained and strained nostrils did not contribute to the classification of the horses in the correct category (“Pain” vs “No Pain”). We compared the LDA to SVM based on a Gaussian Radial Basis kernel with parameter σ = 0.01 and C = 0.75—chosen in order to maximise the LOSOCV accuracy—excluding mouth strained and strained nostrils scores from the training data of SVM, consistently with the LDA weights adopted. Using the same predictive variables, the only difference in the performance of the two methods is due to the methods themselves, and this guarantees a fair comparison between LDA and SVM accuracy. The results showed that in this case LDA and SVM, with a non-linear kernel, had comparable degree of accuracy (77.4% and 77.7% respectively), supporting the use of LDA weights to build an accurate and interpretable classification rule. It is worth noticing that an SVM based on a linear kernel (with C = 0.01) achieved a similar level of accuracy (76.9% computed with LOSOCV procedure), again supporting the use of a linear classifier. Finally, considering the classifier based on the mean of the FAU scores (i.e., using equal weights for every FAU, that is equivalent to using the total MGS score on the six FAUs as the unique predictor), we obtained an accuracy of 72.9% with LOSOCV procedure, lower than using the LDA weights, again supporting the removal of mouth strained and strained nostrils from the FAUs providing information about the horse pain status.

### Mouse Grimace Scale (MGS)

The adjusted p-values of the CLMM are presented in Table 3. When comparing images from pre- to post-procedure, the treatment with Bupivacaine significantly influenced orbital tightening (P = 0.0003) and cheek bulge (P = 0.0003), resulting in statistically higher scores for these two FAUs of the Bupivacaine group with respect to the baseline (“Pre-op” group). The treatment with Meloxicam significantly influenced each FAUs (all p-values below P<0.05), resulting in statistically higher scores for all the FAUs of Meloxicam group, with respect to the baseline. The treatment with Saline significantly influenced all the FAUs (all p-values P<0.0001), resulting in statistically higher scores for all the FAUs of Saline group with respect to the baseline. The observer and the mouse random effects are significantly associated to all the FAUs (all p-values far below P<0.05). Overall, the MGS applied by observers with different background demonstrated an excellent inter-observer reliability with an average ICC value of 0.8405 (95% confidence interval: [0.7380; 0.9165]). ICC values on average scores for each FAU and correspondent 95% confidence intervals are reported in Table 4. Excellent reliability was demonstrated for orbital tightening (ICC = 0. 8580) and ear position (ICC = 0.7560). Cheek bulge showed a good reliability with an ICC of 0.6439. While, fair reliability was demonstrated for the nose bulge (ICC = 0.5571).

**Table 3:** Cumulative Link Mixed Model (CLMM) on MGS dataset. Results from CLMM investigating the effects of Pain treatment (“Pre-op” VS “Post-Bupivacaine”, “Pre-op” VS “Post-Meloxicam” and “Pre-op” VS “Post-Saline”), Observer and Mouse on Facial Action Units. Adjusted p-values with Benjamini-Hochberg correction in relation to the respective category are reported for each FAUs. Correspondent likelihood ratio test statistics of the CLMM on MGS data are reported in brackets.

Facial Action Unit	Pre-op VS Post-Bupivacaine	Pre-op VS Post-Meloxicam	Pre-op VS Post-Saline	Observer	Mouse
Orbital tightening	0.0003 (15.7362)	0.0013 (11.6430)	<0.0001 (202.1082)	<0.0001 (55.4441)	<0.0001 (100.6077)
Ear position	0.2364 (1.4719)	0.0087 (7.2166)	<0.0001 (88.0524)	<0.0001 (45.5261)	<0.0001 (68.8724)
Cheek bulge	0.0003 (14.2784)	0.0003 (13.7758)	<0.0001 (42.6204)	<0.0001 (79.8075)	<0.0001 (21.1832)
Nose bulge	0.4509 (0.5702)	0.0455 (4.1723)	<0.0001 (35.6402)	<0.0001 (111.7367)	<0.0001 (56.4078)

**Table 4:** Results regarding the inter-observer reliability analysis on MGS. Average scores of ICC, with the 95%-confidence interval reported in brackets, are presented for each FAU of MGS. This analysis was not carried out in the Leach et al., 2012 study [18].

Facial Action Unit	Intraclass Correlation Coefficient (ICC)
Orbital tightening	0.8580 (0.7665–0.9259)
Ear position	0.7559 (0.6008–0.8720)
Cheek bulge	0.6439 (0.4267–0.8108)
Nose bulge	0.5572 (0.2971–0.7619)

**Fig 4:**
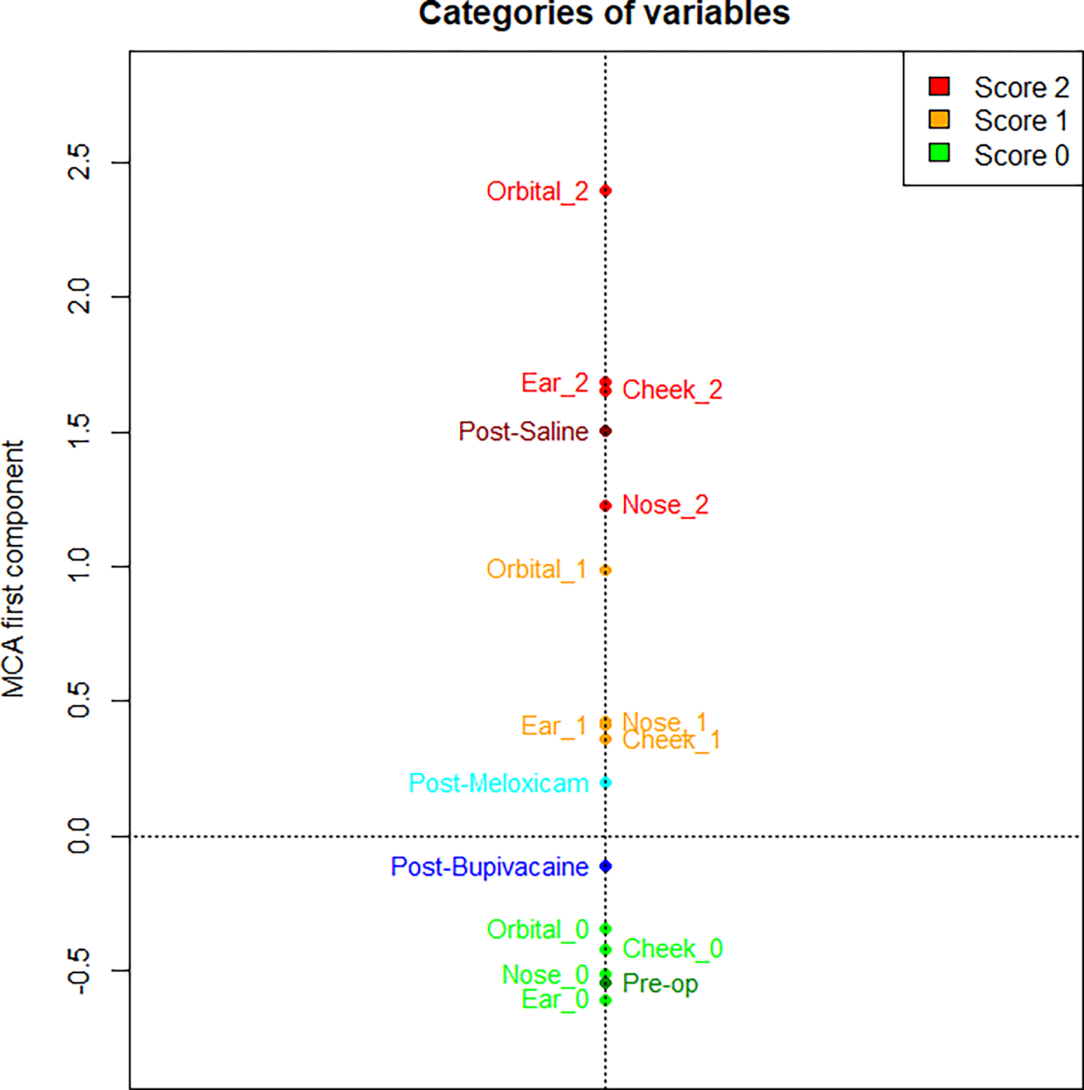
MCA results reported on the first component. The scores (values '0', '1' and '2') of the four FAUs of MGS and the treatment groups (“Pre-op”, “Post-Bupivacaine”, “Post-Meloxicam” and “Post-Saline”) are presented, on average, on the first component of MCA.

**Fig 5:**
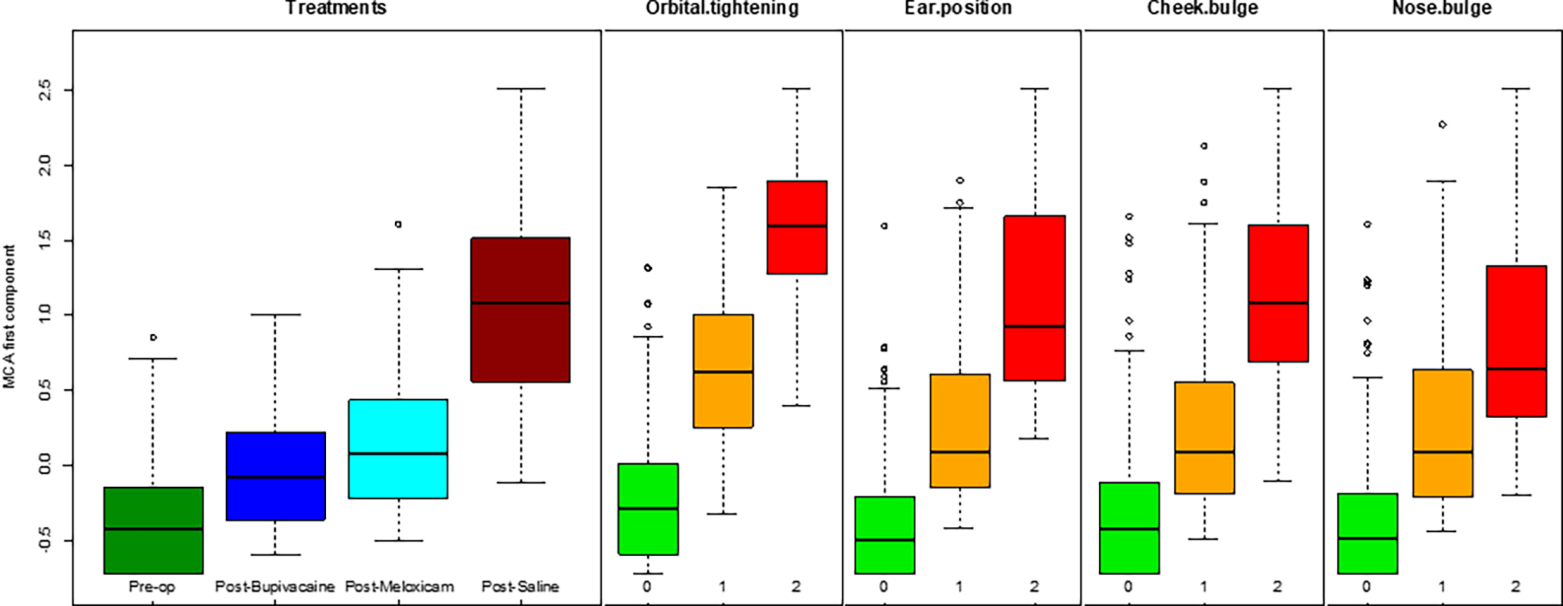
Boxplots of MCA first component scores stratified by treatment group and FAUs score. The boxplots represent the dispersion of the MCA first component scores of the mouse pictures, stratified by treatment group (“Pre-op”, “Post-Bupivacaine”, “Post-Meloxicam” and “Post-Saline”) and by score ("0", "1" and "2") of each FAU.

The MCA of the FAUs scores identified the first component (a one-dimensional space) to accurately describe the co-occurrence of variables, explaining a high proportion of variation (71.76% computed according to Greenacre’s correction). The first MCA component is represented in Fig 4, where we plotted the average value of the component for the images of mice belonging to the same treatment group (e.g., the label “Post-op saline” represents the average position of all the images of mice treated with saline after the operation) and the average value of the component for the images that have the same score for a FAU (e.g. the label “Cheek_2” represents the average position of all the images having score 2 for cheek bulge FAU). This MCA component contrasts categories related to higher level of pain (positive values of the component in Fig 4) with categories related to absence of pain (negative values of the component). The intensity of all four different FAUs are related, meaning that there are three well separated groups of categories, corresponding to the scores. Furthermore, the group “score 0” (i.e. all the labels of FAUs with a score 0 in Fig 4) is related to the category “Pre-op” as they are closer in Fig 4, whereas the group “score 2” is related to the category “Post-Saline”. Categories “Post-Meloxicam” and “Post-Bupivacaine” are between the groups “score 1” and “score 0”, but the former has a positive value, the latter a negative value: “Post-Meloxicam” group is on average more related to higher level of pain with respect to the global mean (located at the origin of axes), instead the “Post-Bupivacaine” group is more related to lower level of pain. The variability of the first component of MCA is reported in Fig 5.

**Fig 6:**
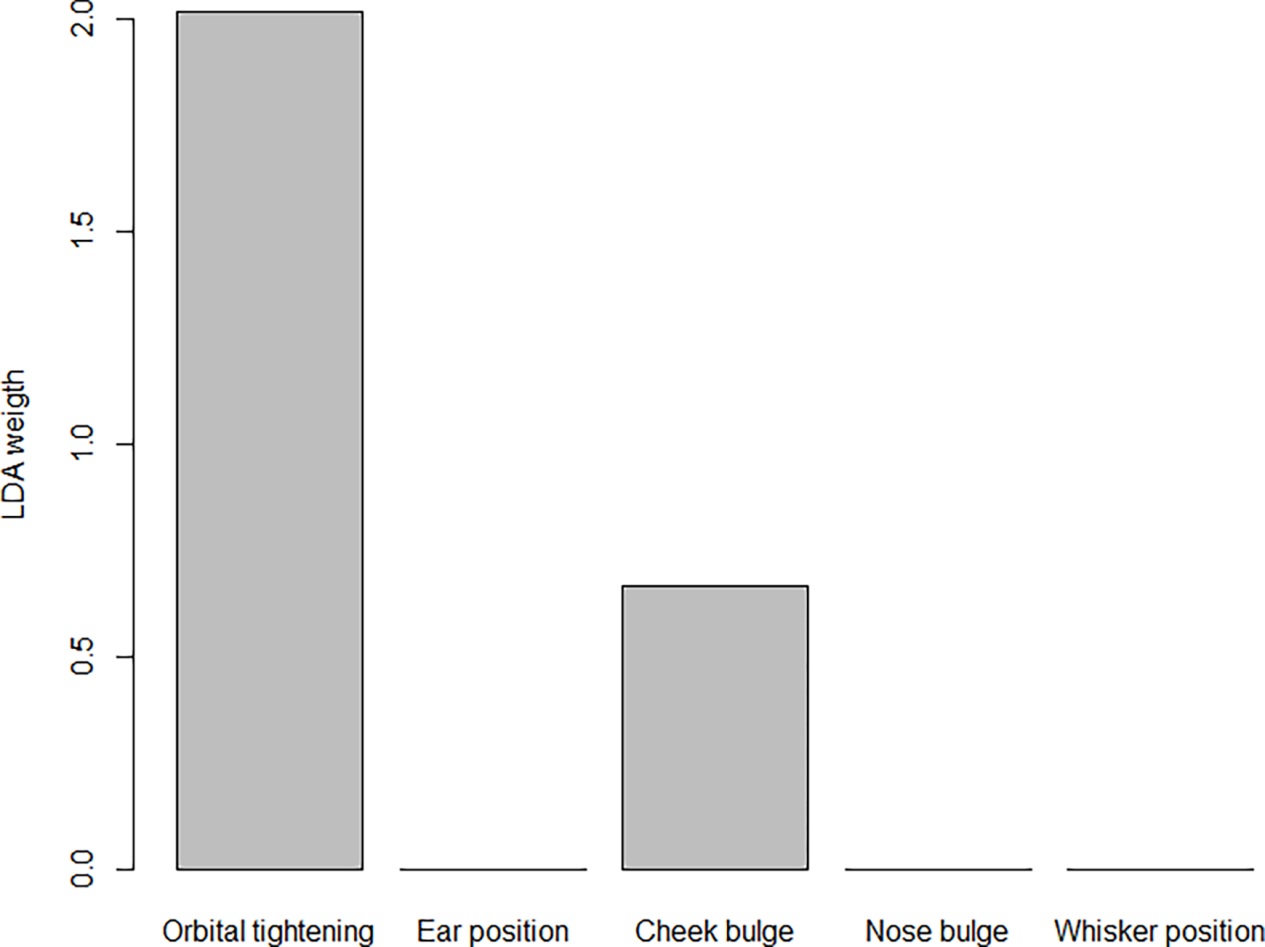
Weights of variables in the LDA component for MGS. Weight for each FAUs of the MGS that best discriminate between the categories (“no pain status” vs “pain status”).

The LDA identified the weights of the FAU scores that can be used to best discriminate the “Pre-op” group (here intended as associated to the “No Pain” status) from the “Post-Saline” (here intended as associated to the “Pain” status) (Fig 6). We did not include the “Post-Bupivacaine” and “Post-Meloxicam” groups in the classification analysis, since from the previous analyses these two groups seemed to lay in between the pain and no pain condition. Among the 429 scores analysed, LDA correctly classified 302 scores related to the “No Pain” group and 72 related to the “Pain” group, while 38 were wrongly assigned to the “No Pain” group and 17 to the “Pain” group. Scores for orbital tightening and cheek bulge contributed to the classification of the mice in the correct category (“Pain” vs “No Pain”). Scores for ear position and nose bulge did not contribute to the classification of the mice in the correct category (“Pain” vs “No Pain”). We compared the LDA to SVM based on a Gaussian Radial Basis kernel with parameter σ = 0.5 and C = 0.75, chosen in order to maximise the LOSOCV accuracy. The results showed that in this case LDA and SVM, with a non-linear kernel, have comparable degree of accuracy (80.1% and 82.3% respectively), supporting the use of LDA weights to build an accurate and interpretable classification rule. It is worth noticing that an SVM based on a linear kernel (with C = 0.01) achieved a similar level of accuracy (80.1% computed with LOSOCV procedure), again supporting the use of a linear classifier. Moreover, considering the classifier based on the sum of the four FAU scores, we obtained an accuracy of 76.3% with LOSOCV procedure, lower than using the LDA weights, again supporting the removal of ear position and nose bulge from the FAUs providing information about the mouse pain status. Finally, when testing the classifier based on LDA weights on the “Post-Bupivacaine” and “Post-Meloxicam” groups, we obtained that 80.2% of the former observations and 81.6% of the latter observations were classified as “No Pain”.

## Discussion

The present study aimed to identify a classifier that is able to estimate the pain status of the animal based on the FAUs included in the HGS and MGS. To achieve this aim a specific statistical approach was applied to published data on the HGS and MGS [18,19], with the hypothesis that pain condition would increase the individual FAU scores. We report three major findings. Firstly, the excellent reliability of both HGS and MGS was confirmed with high values of ICC for almost all the FAUs. Secondly, individual FAU scores of HGS and MGS were related to the pain state of animals. Finally, we identified the optimal weights of the FAU scores that can be used to best classify animals in pain with an accuracy greater than 70%, with the use of LDA.

In the present study, grimace scale scores were assigned by treatment and condition (i.e. time-point) blind observers; therefore, as a first step, checking for inter-observer reliability was paramount. For the HGS, the scores of mouth strained and strained nostrils had the lowest ICC of the six FAUs within this scale: from Table 2, ICC was equal to 0.70 and 0.61 respectively, against ICC values greater than 0.83 for the other FAUs. This suggests that the scores assigned to mouth strained and strained nostrils are more observer-dependent compared to the other FAUs. Moreover, this interpretation is further demonstrated by the significant observer random effect in the CLMM for these two FAUs. Although the ICC for orbital tightening and ear position of MGS was excellent (i.e., high agreement among raters, on average), a significant effect of the observer was found in the same variables (i.e., the measures are observer-dependent). This apparently conflicting result could be explained by fact that the CLMM relates to single ICC, since it evaluates the significance of a single observer with the proper random effect introduced in the model. Here we computed the average ICC, that is known to be higher than single ICC [30], especially with many assessors as in the MGS dataset (21 observers with different levels of experience scoring the pictures). Since the grimace scale scoring relies on observer’s assessment, improving the reliability of all assessors is paramount, especially when they have different backgrounds and knowledge of the species, as in the MGS dataset. A proposed solution is to harmonise the training of assessors (improving the grimace scale manual with higher quality images and drawings, and giving the observers the opportunity to discuss with an experienced observer), and checking their reliability after the training period. Concerning this statistical approach, the average-measures ICC is the most used and reported method to evaluate inter-observer reliability of pain assessment tools [38,39]. Average-measures ICC provides a quantification of the mean of the agreement between observers who make independent scoring using a pain assessment tool, in this case grimace scales. Our results highlight that the use of the CLMM is crucial, as this analysis adds information on how different observers scored the same FAU in the same picture, because of the introduction of the random effect of the observer in the model. Indeed, only the combination of CLMM results with the ICCs enables the researchers to identify both whether and which observers attributed FAU scores quite differently with respect to the other observers. This aspect is important in clinical practice, where new assessors need to be trained in scoring grimace scales to evaluate whether single animals are in pain or not. With this in mind, we suggest that both CLMM and ICC should be analysed in future studies in order to have an overall picture of the reliability of assessors for each FAU.

As a second step, the MCA was used to evaluate the overall relation within the FAU scores, and among the FAUs and the pain condition of the horses, or the treatment of the mice (“Pre-op”, “Post-Bupivacaine”, “Post-Meloxicam” and “Post-Saline”). For both HGS and MGS data, the results of the MCA reveal that categories related to higher level of pain (FAU score = 2) are positioned at opposite sides when compared to categories related to absence of pain (FAU score = 0). When more than one variable is available (e.g. different FAUs), it is critical to define how much each FAU influences the classification process. In horses, there are three well separated and equidistant groups of categories, corresponding to the 3 scores, meaning that on average for each image all the FAUs tend to be scored with the same value (0, 1, or 2). Moreover, Facial Action Units with scores 1 or 2 were more related (closer in Fig 1) to group “Pain”, whereas FAUs with scores 0 were all within group “No Pain”. Interestingly, in mice, not all the treatments influenced the scores of the FAUs in the same way. When mice were treated with saline, the score of each FAUs were significantly higher compared to the baseline; this result confirmed our hypothesis that the MGS and its FAUs were assessing pain. Similarly, the treatment with Meloxicam influenced the scores of all FAUs in the same manner suggesting that this treatment reduced pain only partially. The Bupivacaine group responded differently to the other treatment groups (Saline and Meloxicam) in that only orbital tightening and cheek bulge FAUs increased their scores from pre to post-vasectomy with the other FAUs not changing significantly. This finding could suggest that Bupivacaine is working effectively as it reduces the scores for the other FAUs back towards baseline levels. Further, the significant increase only in orbital tightening and cheek bulge suggests that these FAUs are potentially more sensitive to pain than the other FAUs. Both these FAUs have been shown to be accurate predictors of pain in this and other studies [16–18,23].

Finally, FAUs scores were used to build a classifier that can be used to more accurately recognise animals in pain. Classification, in this case, is the assignment of an observation with some given input values (FAU scores) to one of the output categories (pain or no pain conditions), meaning that applying a classifier enables prediction of the unknown pain condition of an animal, based on a training set of data containing observations whose pain status is known. As a starting point, the CLMM analysis was used to evaluate the validity of the grimace scale scores, in terms of how each FAU’s score was influenced by pain condition (for horses and mice) and treatment (for mice). To date, no studies had taken into account each single FAU in HGS or MGS and how they change in response to pain (or treatment), but this is paramount when the aim is to build a classifier based on the FAUs scores. Our findings show that pain condition affected significantly not only the overall HGS and MGS score, confirming previous findings by Dalla Costa and colleagues [19] and Leach and colleagues [18], but also the individual FAU scores in both horses and mice. This result supports the hypothesis that even single FAUs contribute to pain recognition and response to pain treatment. The CLMM analysis also took in consideration the possible presence of random effects related to the subject itself (horse or mouse) and the observer. In detail, it suggests that the effectiveness of some FAUs scores are dependent on the specific observer (i.e. mouth strained and strained nostrils for horses and all FAUs for mice) and all FAUs scores are dependent on the specific subject. In human medicine, pain perception is well known to be subjective, meaning that the same painful condition in two different subjects can result in two different levels of pain perceived and medication required [40]. Therefore, in clinical settings the concept of the number (of patients) needed to treat (NNT) in order to produce a 50% reduction in pain in one patient was introduced [40]. Data are still not available on individual pain perception in animals, but our results showed that subject itself should be considered when assessing pain, and further studies should be performed to better understand the efficacy of analgesic treatment at individual level.

LDA was then applied with the aim to build a classifier and to identify the weights of the single FAUs in the process of classification. For the HGS, only four FAUs (stiffly backwards ears, orbital tightening, tension above eye area and prominent strained chewing muscles) are needed to build the optimal classifier (the classifier built using all the six FAUs with equal weights indeed performed less well). Interestingly, these FAUs were the most valid and statistically reliable (confirmed by CLMM and ICC results). For the MGS, only orbital tightening and cheek bulge are needed to build the optimal classifier, with the former being definitively the most important one. In both species, the LDA results were similar to SVM, supporting the use of LDA weights to build an accurate classification rule (77.4% and 80.1% for horses and mice respectively).

## Conclusions

For the first time, this study describes a statistical approach to develop a classifier that is able to estimate the pain status based on HGS and MGS. The FAUs weights proposed could be applied in clinical practice with the aim to improve pain assessment and management. The mouse makes an excellent model for human disease, therefore effective pain assessment is not only critical for their welfare, but also for the validity of science using mice as models [18]. On the other hand, horse castration is a husbandry practice routinely performed with 240,000 estimated horses castrated in Europe every year [41], therefore, these results would have a significant impact on equine welfare. A possible limitation of this study is that the proposed weight of the FAUs are based on surgical castration model for horses and vasectomy for mice, so it could be that these weights could vary in other conditions. The classifier proposed here can be considered the starting point to develop a computer-based image analysis technique for the automatic recognition of pain in horses and mice. Further studies should consider increasing the number of high quality pictures and standardize the position of the head of the animal (frontal and/or lateral) in order to simplify the machine learning process, improving the accuracy, as well as other painful conditions (e.g. orthopedic pain) in order to further check the accuracy of the classifier in recognizing pain.

## Supporting information

S1 Table Horse Grimace Scale (HGS) dataset. (TXT)

S2 Table Mouse Grimace Scale (MGS) dataset. (TXT)
